# Middle Meningeal Artery Embolization for the Treatment of Chronic Subdural Hematomas—a German Nationwide Multi-center Study On 718 Embolizations

**DOI:** 10.1007/s00062-025-01549-w

**Published:** 2025-08-18

**Authors:** Dominik F. Vollherbst, Ansgar Berlis, Mahmoud Zaki, Christoph Maurer, Christina Onyinzo, Fee C. Keil, Leonard Mann, Christophe T. Arendt, Marius Hartmann, Steffen Reißberg, Corinna Rutschke, Kai Kallenberg, Stefan Grau, Alexandru Durutya, Adrian Liebert, Heinz L. Voit-Höhne, Markus Holtmannspötter, Christian Herweh, René Chapot, Mohamed Elsharkawy, Dan Meila, Björn Greling, Frederik Boxberg, Dominik Grieb, Cornelius Deuschl, Yahya Ahmadipour, Tobias Boeckh-Behrens, Jannis Bodden, Carsten Lukas, Felix Kämmerer, Daniel Behme, Elie Diamandis, Eberhard Siebert, Aymen Meddeb, Kornelia Kreiser, Sabine Heinz, Stephan Meckel, Semin Berzeg-Kolck, Jens Fiehler, Matthias Bechstein, Marius G. Kaschner, Keihan Darvishi, Laura S. Leukert, Marc A. Brockmann, Thomas E. Mayer, Jan-Hendrik Buhk, Charlotte S. Weyland, Lukas Görtz, Christoph Kabbasch, Werner Weber, Christina Wendl, Tobias Struffert, Christian Dyzmann, Johannes C. Gerber, Martin Bendszus, Markus A. Möhlenbruch

**Affiliations:** 1 Department of Neuroradiology, University Hospital Heidelberg, Heidelberg, Germany; 2 Diagnostic and Interventional Neuroradiology, University Hospital Augsburg, Augsburg, Germany; 3 Neuroradiology, Schön Klinik Vogtareuth, Vogtareuth, Germany; 4 Neurosurgery, Schön Klinik Vogtareuth, Vogtareuth, Germany; 5 Institute of Neuroradiology, University Hospital, Goethe University Frankfurt, Frankfurt am Main, Germany; 6 Institut für Neuroradiologie, Helios Klinikum Berlin-Buch, Berlin, Germany; 7 Institut für Radiologie, Neuroradiologie und Nuklearmedizin, Klinikum Darmstadt, Darmstadt, Germany; 8 Department of Neuroradiology, Klinikum Fulda, Fulda, Germany; 9 Clinic for Neurosurgery, Klinikum Fulda, Fulda, Germany; 10 Department of Neurosurgery, Paracelsus Medical University, Nuremberg, Germany; 11 Department of Neuroradiology, Paracelsus Medical University, Nuremberg, Germany; 12 Department of Neuroradiology, Klinikum Frankfurt Höchst, Frankfurt, Germany; 13 Neuroradiology, Alfried-Krupp-Krankenhaus Rüttenscheid, Essen, Germany; 14 Department of Interventional Neuroradiology, Johanna-Etienne-Hospital, Neuss, Germany; 15 Department of Radiology and Neuroradiology, Sana Kliniken Duisburg, Duisburg, Germany; 16 Department of Diagnostic and Interventional Neuroradiology, Medical School Hannover, Hannover, Germany; 17 Institute for Diagnostic and Interventional Radiology and Neuroradiology, University Medicine Essen, University of Duisburg-Essen, Essen, Germany; 18 Department of Neurosurgery and Spine Surgery, Center for Translational Neuro- and Behavioral Sciences (C-TNBS), University Medicine Essen, University Duisburg-Essen, Essen, Germany; 19 School of Medicine and Health, Institute for Neuroradiology, TUM University Hospital, Technical University of Munich, Munich, Germany; 20 Institute of Neuroradiology, St. Josef-Hospital, Ruhr University Bochum, Bochum, Germany; 21 Clinic for Neuroradiology, University Medical Center Magdeburg, Magdeburg, Germany; 22 Research Campus STIMULATE, University of Magdeburg, Magdeburg, Germany; 23 Institute of Neuroradiology, Charité, Universitätsmedizin Berlin, Berlin, Germany; 24 Department of Neuroradiology, University of Ulm, Ulm, Germany; 25 Institute of Diagnostic and Interventional Neuroradiology, RKH Klinikum Ludwigsburg, Ludwigsburg, Germany; 26 Department of Neuroradiology, Faculty of Medicine, Medical Center-University of Freiburg, University of Freiburg, Freiburg, Germany; 27 Klinik und Poliklinik für Neuroradiologische Diagnostik und Intervention, Universitätsklinikum Hamburg-Eppendorf, Hamburg, Germany; 28 Department of Diagnostic and Interventional Radiology, Medical Faculty, University Dusseldorf, Dusseldorf, Germany; 29 Department of Neuroradiology, University Medical Center Mainz, Johannes Gutenberg University, Mainz, Germany; 30 Sektion Neuroradiologie, Institut für diagnostische und interventionelle Radiologie, Universitätsklinikum Jena, Jena, Germany; 31 Neuroradiology, Asklepios Klinik St. Georg, Hamburg, Germany; 32 Department of Neuroradiology, University Hospital RWTH Aachen, Aachen, Germany; 33 Department of Radiology and Neuroradiology, Faculty of Medicine and University Hospital, University of Cologne, Cologne, Germany; 34 Institut für Diagnostische Radiologie, Neuroradiologie und Nuklearmedizin, Knappschaft Kliniken, Universitätsklinikum Bochum, Bochum, Germany; 35 Department of Radiology, University Medical Center Regensburg, Regensburg, Germany; 36 Center of Neuroradiology, medbo District Hospital and University Medical Center Regensburg, Regensburg, Germany; 37 Department of Neuroradiology, University Hospital Giessen, Giessen, Germany; 38 Neuroradiology Department, Sana Kliniken, Lübeck, Germany; 39 Institute of Neuroradiology, Universitätsklinikum Carl Gustav Carus an der Technischen Universität Dresden, Dresden, Germany

**Keywords:** Middle meningeal artery, Embolization, Chronic subdural hematoma, Chronic subdural hemorrhage

## Abstract

**Background:**

Embolization of the middle meningeal artery (EMMA) is a promising novel technique for the treatment of patients with chronic subdural hematomas (cSDH).

**Methods:**

After a nationwide query in Germany, patients with cSDH, treated with EMMA were retrospectively analyzed. Patient and cSDH characteristics, procedural parameters, complications, and rates of treatment failure (TF; residual cSDH > 10 mm, cSDH progression or requirement of rescue surgery) were investigated. TF rates were compared between first-time treatments and treatments of recurrent cSDH, patients receiving embolization and surgery and those being embolized only, different types of embolic agents (particles vs. liquid agents) and between patients with and without antithrombotic medication.

**Results:**

718 EMMAs (420 unilateral, 149 bilateral) were performed in 569 patients in 30 German neurovascular centers. 57.1% were first-time treatments and 42.9% were treatments of recurrent cSDHs. The most frequently used embolic agents were particles (56.2%), followed by copolymer-based liquid embolic agents (19.6%). The rate of symptomatic procedure-related complications was 2.5%. After a mean follow-up of 6.5 months, TF was observed in 16.2% across all treatments and was more frequent after the treatment of recurrent cSDHs (19.8% vs. 13.5%, *p* = 0.045) and in patients taking antithrombotic drugs (17.7% vs. 11.5%; *p* = 0.044). TF was not significantly different regarding the type of embolic agent or additional surgery.

**Conclusions:**

In this nationwide multi-center study, EMMA was associated with favorable clinical outcomes and a low complication rate, supporting the results of recently published randomized controlled trials. TF was more frequent in recurrent cSDH treatments and in patients taking antithrombotic drugs.

## Introduction

Chronic subdural hematoma (cSDH) is already a very common disease and its incidence will likely rise in the coming years [1]. The standard management of cSDH usually consists of either non-surgical management, including watchful waiting and optimization of risk factors, or surgical management, including craniotomy and burr hole trepanation, with or without the insertion of a drainage system [2]. The main drawback of these standard managements is the high rate of treatment failure (TF), with TF rates of around 50% in case of non-surgical management and around 25% in case of surgical management [3–5]. Inflammatory processes and repetitive, subclinical re-bleedings are believed to play a key role in the persistence and growth of cSDHs [6, 7].

Embolization of the middle meningeal artery (EMMA) was described for the first time by Mandai et al. in 2000 as a technique to break the vicious circle of cSDH persistence and thus to facilitate the physiological resorption of the hematoma [8]. Since then, several studies investigated EMMA and showed that this new technique can be a useful treatment for patients suffering from cSDH [9, 10]. Currently, randomized-controlled trials (RCTs) are being performed, aiming at demonstrating the superiority of the addition of EMMA to standard management compared to standard management alone. The results of the first four RCTs were just recently published, showing promising results and lower TF rates for adjunctive EMMA compared to standard management alone for this new treatment [11–14].

Currently, no standardized treatment strategy exists for the use of EMMA in cSDH, and its application—either as a stand-alone therapy or adjunct to surgery—varies between institutions. A range of embolic agents is employed—most commonly particles, cyanoacrylates, and ethylene-vinyl alcohol copolymer-based materials—with the choice typically guided by operator or institutional preference and anatomical considerations.

The aim of this study was to investigate the current status of EMMA for the treatment of cSDH in Germany and to report current practices and outcomes. Furthermore, this study aims to analyze the difference in TF regarding the treatment of recurrent disease, adjunctive surgical treatment, the choice of embolic agent and the use of antithrombotic medication.

## Materials and Methods

### Study Design

This is a nationwide, multicenter, retrospective, observational study. All academic hospitals and neurointerventional non-academic centers in Germany were contacted and invited to participate. Inclusion criteria were: radiologically confirmed unilateral or bilateral cSDH, endovascular and catheter-based EMMA for the treatment of cSDH, and treatment performed between January 2014 and December 2023. Exclusion criteria were: patient age under 18 years, absence of follow-up imaging (CT or MRI) after treatment, and follow-up imaging performed less than one month after treatment. A survey, designed for this study, was completed by the centers who participated in the study. The clinical and radiological records of patients who received EMMA for the treatment of cSDH were systematically reviewed. Institutional Ethic Committees approved this study and waived the need for written informed consent.

### Patient and cSDH Characteristics

Patient data included age, sex and clinical presentation (symptoms of the cSDH), the modified Rankin Scale (mRS) at presentation and at the follow-up visits and the current medication with a focus on antithrombotic drugs (anticoagulant drugs, antiplatelet drugs or any antithrombotic therapy (anticoagulant and/or antithrombotic drug)). cSDH characteristics included the side of the cSDH, its thickness (maximal thickness, measured in the axial plane) and previous treatments.

### Treatment Characteristics

The recorded treatment characteristics included the access site, the type of anesthesia, type and sub-type of embolic agent, the number and location of embolization positions.

### Complications

Peri- and post-interventional complications were assessed and classified as technical (asymptomatic) and clinical (symptomatic) complications. For clinical complications, relatedness to the procedure and to the embolization device was queried and classified as “not related”, “probably related” and “definitely related”. Serious adverse events (SAEs) were defined as events leading to prolongation of hospital stay, death, life-threatening events or events leading to persistent major disability.

### Outcome

At the latest clinical follow-up, the mRS score was assessed and at the latest radiological follow-up, cSDH size was measured. TF was defined as residual cSDH > 10 mm or hematoma progression at the latest follow-up or requirement of rescue surgery during the follow-up period.

### Statistics

GraphPad Prism (La Jolla, USA; version: 13.3.0) was used for this statistical analysis. Quantitative data are presented as “mean ± standard deviation” or as “absolute number (relative frequency)”. The TF rate was compared between first-time treatments and treatments of recurrent cSDH. To reduce the heterogeneity of data, the following comparisons of TF rates were restricted to patients undergoing first-time treatments: patients receiving embolization and surgery versus those receiving embolization only, embolization using particles versus embolization using liquid embolic agents (LEAs) and patients being under antithrombotic medication at the time of EMMA versus those without antithrombotic medication. Chi-square test was used for statistical comparison. A *p*-value of 0.05 was defined as the threshold for statistical significance.

## Results

**Fig. 1:**
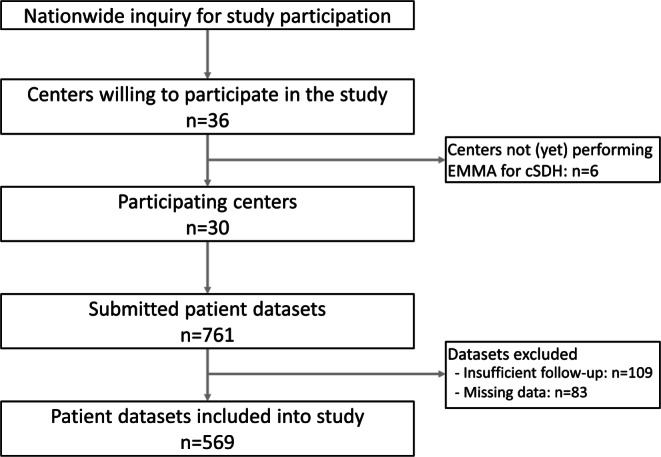
Flowchart illustrating the inclusion of participating centers and enrolled patients. *EMMA* embolization of the middle meningeal artery, *cSDH* chronic subdural hematoma

The inclusion of participating centers and enrolled patients is illustrated in Fig. 1. A total of 36 centers agreed to participate in this study. However, 6 of these centers had not yet treated cSDH patients with EMMA at the time of the data query and did not contribute any data. Consequently, this study included data from 30 German centers, encompassing a total of 569 patients who underwent 718 (420 unilateral, 149 bilateral) EMMA.

### Patient and cSDH Characteristics

Patient, cSDH and treatment characteristics are summarized in Table 1. The mean patient age was 76.3 ± 10.6 years and most patients (77.3%) were male. Pre-treatment mRS was 0 in 67 patients (11.8%), 1 in 138 patients (24.3%), 2 in 141 patients (24.8%), 3 in 148 patients (26.1%), 4 in 66 patients (11.6%) and 5 in 8 patients (1.4%). Anticoagulant drugs were used by 147 patients (25.8%), antiplatelet drugs by 162 patients (28.5%), and any antithrombotic therapy (anticoagulant and/or antithrombotic) by 291 patients (51.1%). Most patients (86.6%) were symptomatic with the following symptoms: headache (58.0%), gait instability (44.9%), motoric deficits (38.0%), seizure (6.7%), vertigo (6.1%), cognitive decline (6.1%) and aphasia (5.2%).

**Table 1:** Patient, cSDH and treatment characteristics

*Antithrombotic medication*	Anticoagulant drugs 147 (25.8%)		Antiplatelet drugs 162 (28.5%)		Any anti- thrombotic drug 291^1^ (51.1%)		No anti- thrombotic drug 278 (48.9%)	
*Symptoms*	Headache 278 (58.0%)	Gait instability 215 (44.9%)	Motoric deficits 182 (38.0%)	Seizure 32 (6.7%)	Vertigo 29(6.1%)	Cognitive decline 29 (6.1%)	Aphasia 25 (5.2%)	Asymp- tomatic 74 (13.4%)
*Location of cSDH*	Right 220 (39.2%)			Left 205 (36.5%)			Bilateral 137 (24.4%)	
*First-time treatment* *or treatment of* *recurrent cSDH*	First-time treatment 337 (57.1%)				Treatment of recurrent cSDH 253 (42.9%)			
*Embolic agent*	Particles 382 (59.8%)	Particles +Coils 85 (13.3%)	Onyx 76 (11.9%)	Squid 44 (6.9%)	PHIL 13 (2.0%)	Cyano- acrylates 39 (6.1%)	Coils 33 (5.2%)	Others 7 (1.1%)
*Particle size*	45–150 µm 145 (32.8%)		100–300 µm 226 (51.1%)		250–355 µm 32 (7.2%)		300–500 µm 39 (8.8%)	
*MMA branch selected for embolization*	Main trunk 333 (28.5%)		Frontal branch 443 (33.1%)				Parietal branch 387 (28.5%)	
*Catheter position in the selected branch*^*2*^	Proximal third 761 (60.5%)		Middle third 204 (24.6%)				Distal third 124 (14.9%)	

Data indicated as Mean ± standard deviation or absolute number of cases (relative frequency in %)

^1^ 18 patients were using anticoagulant *and* antithrombotic medication

^2^ the selected arterial branch was divided into proximal, middle, and distal thirds, and the embolization site was classified accordingly

39.2% cSDHs were right-sided, 36.5% left-sided and 24.4% were bilateral. The mean diameter was 16.7 ± 6.9 mm. 57.1% were first-time treatments and 42.9% were treatments of recurrent cSDHs with previous surgical treatment.

### Treatment Characteristics

EMMA was performed in combination with surgery in 59.2% of cases, predominantly prior to EMMA (87.9%) and less frequently afterward (12.2%). In 40.8% of cases, EMMA was performed alone without additional surgical treatment.

In the vast majority of cases (97.9%), femoral access was used, while radial access was only chosen in 2.1%. Patients were treated under general anesthesia in 57.5%, under local anesthesia in 39.5% and under conscious sedation in 3.0%. The embolic agents used were as follows: particles in 59.8%, particles combined with coils in 13.3%, copolymer-based LEAs in 20.8% (Onyx (Medtronic Neurovascular, Irvine, USA): 11.9%, Squid (Balt, Montmorency, France): 6.9%, PHIL (Terumo Neuro, Aliso Viejo, USA): 2.0%), cyanoacrylates in 6.1%, coils alone in 5.2%, and other embolic agents in 1.1%. The most frequently used particle sizes were 100 to 300 µm in 51.1%, 45 to 150 µm in 32.8%, 300 to 500 µm in 8.8% and 250 to 355 µm in 7.2%. Low-viscosity agents (Squid 12 and PHIL LV) were used in 26.3% of copolymer-based LEA cases. The MMA branch selected for embolization was the frontal branch in 37.9% of cases, parietal branch in 33.1% and the main trunk in 28.5%. The mean length of hospital stay was 10.6 ± 11.0 days.

### Adverse Events and Complications

Adverse events and complications are summarized in Table 2. Technical (asymptomatic) complications were reported in 1.5% (related to the number of embolizations) and included the following: non-target embolization (*n* = 4), vessel perforation (*n* = 3), catheter rupture (*n* = 3) and vasospasm (*n* = 1).

**Table 2:** Adverse events and complications

* Clinical (symptomatic) complications*^*1*^ 19 (3.3%)			
*Relatedness to procedure*^*1*^	Definitely related 4 (0.7%)	Possibly related 11 (1.9%)	Not related 6 (1.1%)
*Relatedness to embolization device*^*1*^	Definitely related 2 (0.4%)	Possibly related 3 (0.5%)	Not related 14 (2.5%)
*Outcome of complication*^*2*^	Completely recovered 16 (84.2%)	Partially recovered 2 (10.5%)	Ongoing 1 (5.3%)

Data indicated as absolute number of cases (relative frequency in %). Relative frequency is indicated ^1^ related to the number of patients or ^2^ related to the number of clinical complications.

Clinical (symptomatic) complications are summarized in more detail in Table 3. The overall clinical complication rate was 3.3% (related to the number of patients). The most common clinical complications were seizures (*n* = 4), ischemic infarctions (*n* = 2), confusion (*n* = 2) and groin hematomas (*n* = 2). Clinical complications being possibly or definitely related to the procedure were reported in 2.5% and clinical complications being possibly or definitely related to the embolization device in 0.9%. SAEs were reported in 1.2% of cases, with only 3 SAEs (0.5%) being possibly or definitely related to the procedure. In one of these patients, coiling of the proximal MMA was performed via a recurrent meningeal artery variant (MMA origin from the ophthalmic artery). Despite an uneventful and technically successful procedure, the patient developed a scotoma caused by a retinal artery branch occlusion from which she partially recovered. The other SAEs with relation to the embolization procedure were a case of infected groin hematoma, which led to a prolongation of the hospital stay, and a case of persistent post-treatment confusion with no evident underlying cause. Most patients (84.2%) recovered from their complications, while partial recovery was observed in 10.5% and ongoing symptoms in 5.3%.

**Table 3:** Clinical adverse events

Adverse event (AE)	Treatment of AE	Relatedness to embolization procedure	Relatedness to embolization device	Serious AE?	Outcome
Seizure	Reanimation, anticonvulsive medication	Not related	Not related	Yes	Partially recovered
Seizure	Anticonvulsive medication	Possibly related	Not related	No	Completely recovered
Seizure	Anticonvulsive medication	Possibly related	Not related	No	Completely recovered
Seizure	Anticonvulsive medication	Possibly related	Not related	No	Completely recovered
Ischemic stroke^1^	None	Possibly related	Possibly related	No	Completely recovered
Ischemic stroke^2^	Aspirin	Possibly related	Possibly related	No	Completely recovered
Confusion	None	Possibly related	Not related	No	Completely recovered
Confusion	None	Possibly related	Not related	Yes	Ongoing
Infected groin hematoma	Surgery, antibiotic treatment	Definitely related	Not related	Yes	Completely recovered
Groin hematoma	Conservative	Definitely related	Not related	No	Completely recovered
Aphasia	None	Possibly related	Not related	No	Completely recovered
Aphasia^3^	Surgery	Not related	Not related	No	Completely recovered
Headache^3^	None	Possibly related	Not related	No	Completely recovered
Headache	None	Possibly related	Possibly related	No	Completely recovered
Palsy of abducens nerve	None	Definitely related	Definitely related	No	Completely recovered
Retinal artery branch occlusion	None	Definitely related	Definitely related	Yes	Partially recovered
Chest pain	Cardiac stent implantation	Not related	Not related	Yes	Completely recovered
Pneumonia	Antibiotic treatment	Not related	Not related	Yes	Completely recovered
Infection of cSDH after surgery	Antibiotic treatment	Not related	Not related	Yes	Completely recovered

^1^Infarction in the head of the caudate nucleus

^2^Small infarction in the territory of the anterior cerebral artery

^3^Due to mass effect of the cSDH

### Outcome

**Fig. 2:**
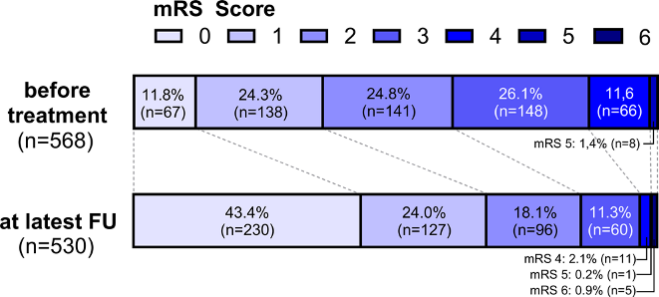
Distribution of mRS scores before treatment and at latest FU. Information on mRS at latest FU was missing in 38 patients. Five patients (0.9%) died during the FU period unrelated to the cSDH

Outcome parameters are summarized in Table 4. Information on mRS, radiological follow-up and TF was available in 531/569 patients (93.3%), 569/569 patients (100%) and 569/569 patients (100%), respectively. After a mean clinical follow-up of 9.0 months, mRS improved in 41.1%, was stable in 54.5% and worsened in 4.4% (mRS distribution illustrated in Fig. 2). Five patients (0.9%) died during the FU period unrelated to the cSDH. After a mean radiological follow-up of 6.5 months, absolute cSDH size reduction was 13.0 ± 7.3 mm and relative size reduction was 76.4 ± 7.3%. Complete hematoma resolution was observed in 46.8%. Regarding all treatments, residual hematoma > 10 mm was reported in 9.1% and progredient hematoma in 1.8%, while rescue surgery was required in 8.2%, resulting in an overall TF rate of 16.2%. Regarding only first-time treatments, residual hematoma > 10 mm was reported in 6.4% and progredient hematoma in 1.2%, while rescue surgery was required in 7.9%, resulting in a first-time treatment TF rate of 13.5%.

**Table 4:** Outcome and statistical analyses

**Radiological outcome and treatment failure**			
*cSDH size reduction*	Absolute size reduction 13.0 ± 7.3 mm	Relative size reduction 76.4 ± 7.3%	Complete hematoma resolution 266 (46.8%)
*Treatment failure*	Residual hematoma > 10 mm 52 (9.1%)	Progredient hematoma 10 (1.8%)	Requirement of rescue surgery 49 (8.2%)
	Overall treatment failure (residual hematoma, progredient hematoma and/or rescue surgery) 92 (16.2%)		
**Statistical analyses**			
*First-time treatment vs. treatment of recurrent cSDH*	TF first-time treatment 44 (13.5%)	TF treatment recurrent cSDH 48 (19.8%)	*p* = 0.045
*Particles vs. liquid embolic agents*	TF particles 25 (14.4%)	TF liquid embolic agents 9 (13.0%)	*p* = 0.788
*Any antithrombotic medication vs. no antithrombotic medication*	TF any antithrombotic medication 29 (16.4%)	TF no antithrombotic medication 13 (8.7%)	*p* = 0.040

Data indicated as mean ± standard deviation or absolute number of cases (relative frequency in %). Relative frequency is indicated related to the number of patients for “cSDH size reduction” and “Treatment failure” and to the number of patients in the respective sub-group for the statistical analyses.

### Comparative Analyses

The results of the comparative analyses are summarized in Table 3. TF was significantly more frequent in patients who were treated for recurrent disease than in those treated for the first time (13.5% vs. 19.8%, *p* = 0.045). No statistical difference was found between patients receiving embolization and surgery and those being embolized only (embolization and surgery: 12.9%, embolization only: 13.1%, *p* = 0.960). Also, comparing patients treated with particles and those treated with LEAs, no statistical difference in TF rate was found (particles: 14.4% vs. LEAs: 13.0%, *p* = 0.788). Regarding the use of antithrombotic drugs, the rate of TF was significantly higher in patients taking those drugs at the time of EMMA compared with those without antithrombotic medication (17.7% vs. 11.5%; *p* = 0.044).

## Discussion

In this nationwide, retrospective multi-center study, EMMA was an effective and safe treatment for patients suffering from cSDH. With 569 patients, treated with 718 EMMA, this is one of the largest studies reporting on this new technique.

Two of the four recently published RCTs observed significantly lower rates of TF for patients treated with EMMA additionally to standard management, compared to standard management alone: [11, 12] In the EMBOLISE study [11], the TF rate was 4.1% in the embolization group and 11.3% in the standard management group, and in the STEM study [12], the rate of TF was 15.8% in the embolization group, as compared with 36.4% in the standard management group. The MAGIC-MT trial [13], however, reported similar rates of TF between treatment and control group (6.7% vs. 9.9%), but EMMA was associated with a lower incidence of SAEs. Also, the most recently published RCT EMPROTECT did not observe a significant difference in the primary endpoint between the embolization group and the control group (14.5% vs. 21.0%). [14] Despite two of the four RCTs not demonstrating a statistically significant difference in their respective primary endpoints, both trials nevertheless showed numerical trends favoring embolization over standard management, suggesting a potential clinical benefit despite the lack of statistical significance. Given this evidence, the indication for EMMA in cSDH patients is likely to expand in the near future, and further research in this field is needed.

TF is the primary outcome parameter in the aforementioned RCTs and also in the vast majority of comparative studies published on EMMA for cSDH in the past. The definition of this crucial parameter is similar but not identical across the literature. The definition of TF in our study (residual cSDH > 10 mm, hematoma progression, or requirement of rescue surgery) was chosen to align with definitions in the recently published RCTs. We specifically determined the rate of TF for patients with first-time cSDH treatments (i.e., excluding those with previous surgery or embolization) to reduce the heterogeneity of our data and for better comparison with the RCTs and previous works. This first-time treatment TF rate of 13.5% in our study is in line with the results of the published RCTs (TF rates: 4.1% in EMBOLISE, 15.8% in STEM, 6.7% in MAGIC-MT and 14.8% in EMPROTECT), although it should be noted that TF was not uniformly defined across the RCTs. We therefore also used the sub-group of patients with first-line treatments for comparative statistical analyses.

Although functional independence of patients, as measured by the mRS, is not the primary endpoint in studies on the treatment of cSDH, it should be emphasized that our study observed a substantial improvement in functional independence after treatment. For example, the rate of mRS 0–2 increased from 60.9% before treatment to 85.5% after treatment. It is important to note, however, that the observed improvement in mRS can largely be attributed to the effects of surgical intervention (if performed), the natural course of the disease, and supportive medical and rehabilitative care, with embolization contributing as an adjunctive factor.

Only limited research is available regarding the comparison of patients receiving first-time cSDH treatments with EMMA and those being treated for recurrent cSDH [15, 16]. We observed a significantly higher rate of TF in patients who have already been treated surgically (19.8% vs. 13.5%). This tendency was also observed by Salah et al. who analyzed 149 surgically treated cSDHs of which 25.5% had undergone prior surgical intervention and found a TF rate of 11% for previously treated cSDHs vs. only 7% in cSDHs without prior surgery [16]. This difference can be explained by pathophysiological mechanisms which are likely more pronounced in recurrent cSDHs, such as inflammatory processes, formation of membranes being supplied by fragile blood vessels which are prone to hemorrhage. Another possible explanation for this finding is that not all branches of the MMA might be patent anymore after a previous surgical intervention, which could impede effective embolization of the cSDH. Further studies should focus on these two patient groups, particularly since patients with recurrent disease were largely excluded from the RCTs.

A frequently debated topic is the selection of the most suitable embolic agent for EMMA. While particles are widely available and cost-effective, LEAs are often credited with potential advantages due to their better visibility, which enables improved control and thus safety, as well as their potentially deeper penetration into the MMA. Efficacy and safety were shown for all types of embolic agents (particles, LEAs and also for coils) in several non-randomized studies [17–19]. In three of four of the recently published RCTs, the LEAs Onyx and Squid were used, while in several RCTs, which are still ongoing or not yet published, EMMA was performed using other embolic agents too, such as particles and cyanoacrylates. EMPROTECT, the first published RCT which used particles, did not observe a statistical difference in the primary endpoint [14]. Based on the available RCT data, no definitive conclusions can be drawn regarding the superiority of any particular embolic agent. Future randomized controlled trials directly comparing embolic agents, as well as meta-analyses of existing and forthcoming RCTs, are needed to address this question. In our study, we compared the TF rates of patients treated with particles and LEAs and did not find a significant difference between those groups which is in line with the results of most non-randomized studies focusing on this topic [20–23].

The use of antithrombotic drugs is a known risk factor for the development of cSDHs [24]. The role of antithrombotic medication and the management of patients who are dependent on these drugs is a frequently discussed topic. It was already shown that antithrombotic drug use increases the rate of recurrence after cSDH drainage without additional embolization [25, 26]. In our study on patients treated by EMMA (with and without additional surgery), we analogously observed a significantly higher TF rate of 17.7% in patients under antithrombotic therapy versus only 11.5% in those without any antithrombotic drug.

Previously, official guidelines for the management of patients with cSDH were lacking. Recent guidelines and consensus papers aim at proposing treatment regimens and defining the role of EMMA [27, 28]. Surgery is generally indicated in patients with symptomatic cSDHs and in cases of large hematomas (> 10 mm) with relevant midline-shift (> 5 mm) being an important imaging marker for decision-making [29]. We did not observe a difference in TF rates between patients who were treated by EMMA alone and those who received EMMA in addition to surgery. Nevertheless, surgery should always be performed when clinically indicated, as embolization is currently not recommended as a replacement for surgical therapy in symptomatic patients or those with large hematomas. However, our findings support the role of “stand-alone” EMMA in selected patients. EMMA without additional surgery may be considered for patients who are not surgical candidates and for those with minimally symptomatic hematomas.

This study has several limitations that should be considered when interpreting the findings. The self-adjudicated data entry and retrospective design may introduce biases related to data collection and analysis. Additionally, the inclusion of diverse surgical techniques and embolic agents, along with variability in whether patients underwent surgery in addition to embolization, may influence direct comparisons and thus TF rates. For statistical analyses, LEAs were grouped together due to their similar properties. However, it is important to note that any inherent differences between these agents could represent a potential source of bias in the results of the comparative analyses. The follow-up duration, with a mean of 6.5 months, limits the assessment of long-term outcomes, such as the risk of recurrence or delayed complications. Furthermore, the absence of a control group without embolization limits the ability to draw definitive conclusions about the efficacy of EMMA. To address these limitations, future prospective and randomized studies with standardized protocols and extended follow-up periods are necessary.

## Conclusion

This large, nationwide study found that EMMA was associated with favorable clinical outcomes, substantial hematoma reduction and a low complication rate in patients with cSDH. Treatment was less effective in recurrent cSDHs and in patients on antithrombotic medications, emphasizing the need for tailored approaches in those populations. TF rates were similar regarding the type of embolic agent and additional surgery. These findings contribute to the growing body of evidence supporting EMMA as a valuable adjunct or alternative to traditional management methods, while highlighting areas for further research.
